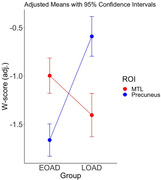# Dissociable spatial topography of neurodegeneration in Early‐onset and Late‐onset Alzheimer’s Disease: A head‐to‐head comparison of MRI‐derived atrophy measures between the LEADS and ADNI cohorts

**DOI:** 10.1002/alz.091818

**Published:** 2025-01-09

**Authors:** Yuta Katsumi, Alexandra Touroutoglou, Michael Brickhouse, Ryan Eckbo, Renaud La Joie, Ani Eloyan, Kelly N. Nudelman, Tatiana M. Foroud, Jeffrey L. Dage, Maria C. Carrillo, Gil D. Rabinovici, Liana G. Apostolova, Bradford C. Dickerson, the LEADS Consortium

**Affiliations:** ^1^ Frontotemporal Disorders Unit and Massachusetts Alzheimer’s Disease Research Center, Department of Neurology, Massachusetts General Hospital and Harvard Medical School, Boston, MA USA; ^2^ Massachusetts General Hospital and Harvard Medical School, Boston, MA USA; ^3^ Memory and Aging Center, Weill Institute for Neurosciences, University of California, San Francisco, San Francisco, CA USA; ^4^ Department of Biostatistics, Brown University, Providence, RI USA; ^5^ Department of Medical and Molecular Genetics, Indiana University School of Medicine, Indianapolis, IN USA; ^6^ Department of Neurology, Indiana University School of Medicine, Indianapolis, IN USA; ^7^ Alzheimer's Association, Chicago, IL USA; ^8^ Department of Radiology and Imaging Sciences, Indiana University School of Medicine, Indianapolis, IN USA

## Abstract

**Background:**

Understanding how early‐onset Alzheimer’s disease (EOAD) differs from typical late‐onset AD (LOAD) is an important goal of AD research that may help increase the sensitivity of unique biomarkers for each phenotype. Building upon prior work based on small samples, here we leveraged two large, well‐characterized natural history study cohorts of AD patients (LEADS and ADNI3) to test the hypothesis that EOAD patients would show more prominent lateral and medial parietal and lateral temporal cortical atrophy sparing the medial temporal lobe (MTL), whereas LOAD patients would show prominent MTL atrophy.

**Method:**

We investigated differences in the spatial topography of cortical atrophy between EOAD and LOAD patients by analyzing structural MRI data collected from 211 patients with sporadic EOAD and 88 cognitively unimpaired (CU) participants from the LEADS cohort as well as 144 patients with LOAD and 365 CU participants from the ADNI3 cohort. MRI data were processed via FreeSurfer v6.0 to estimate cortical thickness for each participant. A direct comparison of cortical thickness was performed between EOAD and LOAD patients based on W‐scores (i.e., Z‐scores adjusted for age and sex relative to CU participants within each cohort) while controlling for MMSE total scores. All patients underwent amyloid PET with ^18^F‐Florbetaben or ^18^F‐Florbetapir and amyloid positivity was centrally determined by quantification‐supported visual read.

**Result:**

As expected, a direct comparison of cortical thickness between patients with EOAD and LOAD revealed a double dissociation between AD clinical phenotype and localization of cortical atrophy: EOAD patients showed greater atrophy in widespread cortical areas including the inferior parietal lobule (EOAD marginal mean W‐score ± SEM = ‐1.33±0.08 vs. LOAD = ‐0.52±0.09, p<.001, η^2^=.097), precuneus (‐1.66±0.09 vs. ‐0.59±0.10, p<.001, η^2^=.13), and caudal middle frontal gyrus (‐1.65±0.08 vs. ‐0.90±0.10, p<.001, η^2^=.074), whereas LOAD patients showed greater atrophy in the entorhinal/perirhinal cortex and temporal pole (‐1.00±0.09 vs. ‐1.41±0.11, p<.008, η^2^=.019).

**Conclusion:**

These findings demonstrate a clearly dissociable spatial pattern of neurodegeneration between EOAD and LOAD, supporting our previously developed LOAD and EOAD signatures of cortical atrophy, which underlies the distinct episodic memory and other cognitive characteristics of these AD clinical phenotypes.